# Self-Expandable Transcatheter Aortic Valves in Patients With Small Aortic Annulus: The SWEDEHEART Registry

**DOI:** 10.1016/j.shj.2025.100680

**Published:** 2025-06-18

**Authors:** Antros Louca, Anna Myredal, Monér Alchay, Henrik Hagström, Dan Ioanes, Stefan James, Sasha Koul, Araz Rawshani, Björn Redfors, Andreas Rück, Kristofer Skoglund, Sebastian Völz, Petur Petursson, Truls Råmunddal, Oskar Angerås

**Affiliations:** a Department of Molecular and Clinical Medicine, Institute of Medicine, University of Gothenburg, Gothenburg, Sweden; b Department of Cardiology, Sahlgrenska University Hospital, Gothenburg, Sweden; c Department of Public Health and Clinical Medicine, Umeå University, Umeå; d Heart Centre, Umeå University Hospital, Umeå, Sweden; e Department of Medical Sciences, Cardiology, Uppsala University, Uppsala, Sweden; f Department of Clinical Sciences, Cardiology, Lund University Hospital, Lund, Sweden; g Department of Cardiology, Karolinska University Hospital and Karolinska Institute, Stockholm, Sweden

**Keywords:** Aortic valve stenosis, Self-expandable valves, Small aortic annulus, Transcatheter aortic valve replacement

## Abstract

**Background:**

Small aortic annulus is associated with poorer hemodynamic outcomes in transcatheter aortic valve replacement (TAVR). In these cases, supra-annular (SA) self-expandable valves (SEVs) may offer better results than intra-annular SEVs (IA SEVs). This study evaluated clinical and hemodynamic outcomes for SA SEVs (Evolut valves, Acurate valves) and IA SEVs (Portico/Navitor valves).

**Methods:**

We analyzed data from patients who underwent TAVR in Sweden between 2013 and 2022 with an annular diameter ≤23 mm, using inverse probability of treatment weighting. Endpoints included mortality, device, and technical success as per Valve Academic Research Consortium 3 definitions. Other endpoints were the incidence of post-TAVR mean or peak gradients over 20 and 40 mmHg, respectively, significant paravalvular leakage, new pacemaker implantation, and postprocedural aortic valve gradients.

**Results:**

The study included 1068 patients, with a median age of 81.2 years, and 88% were women. After inverse probability of treatment weighting adjustment, no differences were observed in the outcomes apart from a marginally lower risk of postprocedural mortality in the Portico/Navitor valves compared to the Evolut valves (adjusted odds ratio: 0.99; *p* ​= ​0.05; 95% CI: 0.98-1.00). Hemodynamically, the Evolut valves showed the lowest mean gradients, followed by the Portico/Navitor valves and the Acurate valves (7.97 vs 9.02 mmHg vs. 0.84 mmHg, respectively, *p* ​< ​0.001; 95% CI: 0.35-1.00).

**Conclusions:**

SA and IA SEVs show comparable clinical outcomes and excellent hemodynamic performance in patients with small aortic annuli. Further studies, including randomized trials, are needed to provide clearer guidance on valve selection.

## Highlights

• Small aortic annulus affects outcomes after transcatheter valve replacement.
• Self-expanding valves show better hemodynamics than balloon-expandable valves.
• Clinical outcomes are similar between intra- and supra-annular valve designs.
• Evolut valves exhibit the lowest gradients, followed by Abbott Portico/Navitor valves.
• Careful valve choice in patients with small aortic annuli is indicated.

## Introduction

Transcatheter aortic valve replacement (TAVR) has emerged as the predominant treatment for severe aortic stenosis.^1^^,^^2^ Currently, 3 primary types of prosthetic valves are utilized in clinical practice: supra-annular self-expanding valves (SA SEVs), intra-annular SEVs (IA SEVs), and IA balloon-expandable valves (IA BEVs).^3^, ^4^, ^5^ The importance of forward-flow hemodynamics following aortic valve replacement has been recognized for decades, and previous research has demonstrated that a postprocedural mean gradient exceeding 20 mmHg after TAVR is associated with higher 4-year mortality.^6^^,^^7^ Despite the increasing number of TAVR procedures, few comparative studies evaluate the different types of SEVs.

Prior research has indicated that SA SEVs have superior hemodynamic outcomes compared to BEVs, particularly in patients with smaller anatomies.^8^^,^^9^ The self-expanding or balloon-expandable TAVR in patients with small aortic annulus (SMART) trial^10^ specifically compared the Evolut valves (SA SEV) to the SAPIEN valves (IA BEV) in patients with small aortic annuli. The study concluded that the Evolut valves were noninferior to the SAPIEN ones concerning the clinical composite endpoint comprising death, disabling stroke, or rehospitalization for heart failure at 12 months. Furthermore, at 1 ​year, the Evolut valve demonstrated superiority over the SAPIEN in the bioprosthetic valve dysfunction endpoint.

Unique characteristics among different transcatheter heart valves (THVs) may influence hemodynamic performance, potentially affecting short- and long-term prognosis. Given the limited data available, this study aims to compare an IA SEV THV with contemporary SA SEVs regarding mortality outcomes, device success, technical success, and hemodynamic performance.

## Methods

### Study Design and Data Sources

In this retrospective observational study, the primary study population was identified from the SWENTRY registry (SWEdish traNscatheter cardiac intervention regisTRY), encompassing patients who underwent TAVR between January 1, 2013, and December 31, 2022. SWENTRY is a part of the SWEDEHEART initiative (Swedish Web-system for Enhancement and Development of Evidence-based Care in Heart Disease Evaluated according to Recommended Therapies)^11^ It provides comprehensive nationwide coverage of the TAVR population in Sweden.^12^ All available variables were extracted, including demographic characteristics, diagnostic information, laboratory values, intraprocedural and postprocedural data, and mortality data.

### Patient Selection

**Figure 1:**
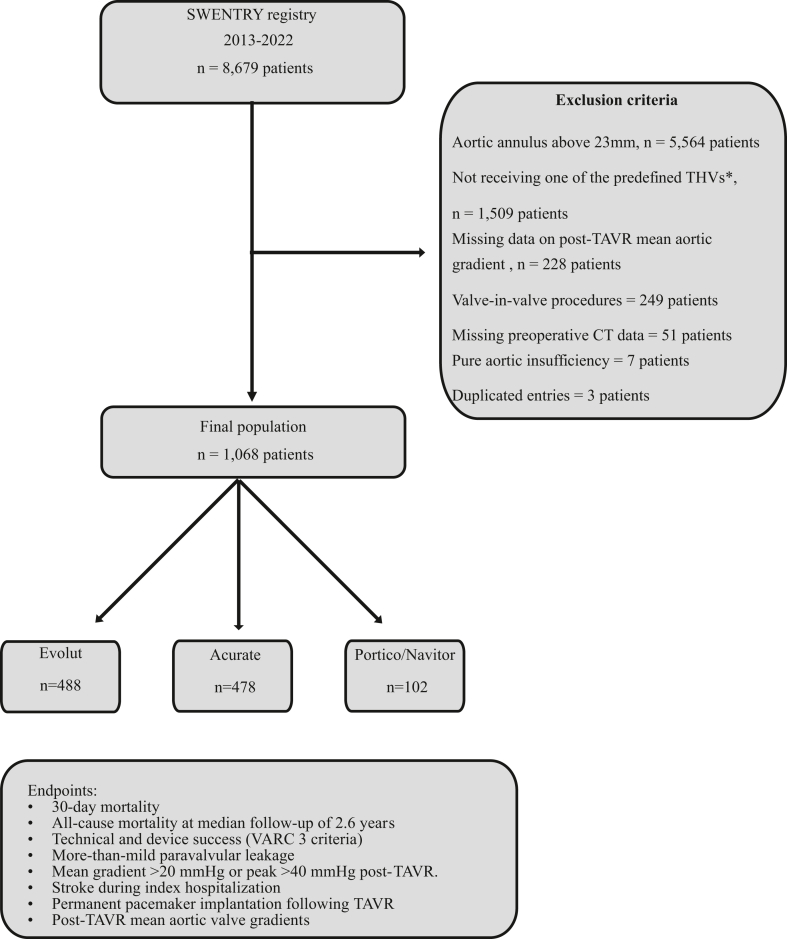
Patient selection process. ∗Only Evolut valves, Portico/Navitor valves, and Acurate vaves were included. Abbreviations: CT, computed tomography; SWENTRY registry, SWEdish traNscatheter cardiac intervention regisTRY; TAVR, transcatheter aortic valve replacement; THVs, transcatheter heart valves; VARC, Valve Academic Research Consortium.

Inclusion criteria included patients with small aortic annuli, defined by a preoperative electrocardiogram-gated computed tomography measurement of ≤23 mm in perimeter-derived mean diameter, with severe aortic stenosis who were treated with second or later iterations of SA SEVs (Evolut and Acurate valves) and IA SEVs (Portico/Navitor valves). Patients with both tricuspid and bicuspid morphologies were included. Exclusion criteria comprised valve-in-valve procedures, either previous TAVR or surgical aortic valve replacement, the absence of preoperative computed tomography data, or missing data on post-TAVR mean and peak gradients (Figure 1 for patient selection).

### Outcomes

The main outcomes were mortality endpoints, including 30-day mortality and all-cause mortality for the median follow-up time of 969 days (interquartile range [IQR]: 478-1452 days), along with technical and device success during the index hospitalization as defined by the Valve Academic Research Consortium 3 criteria^13^ (Supplementary Table 1 for definitions).

Other outcomes included the occurrence of a postoperative mean gradient exceeding 20 mmHg or a peak gradient over 40 mmHg, more-than-mild paravalvular leakage (PVL) during the index hospitalization, the incidence of pacemaker implantation post-TAVR, and the mean aortic valve gradient post-TAVR. More-than-mild PVL was defined using a three-grade classification scheme (mild, moderate, severe) based on postoperative echocardiography, employing both semiquantitative and quantitative parameters as outlined by the Valve Academic Research Consortium 3 criteria.^13^

### Statistical Analysis

Continuous variables are presented as mean ​± ​SD unless otherwise stated. For comparisons of continuous variables between 2 groups, the Welch’s *t*-test was used. The Welch’s F-test (Welch’s analysis of variance) was applied for continuous variable comparisons across more than 2 groups. If Welch’s F-test indicated statistical significance, post hoc pairwise comparisons were performed using the Games-Howell test. Categorical variables are expressed as counts and percentages, with group differences assessed using the chi-square test or Fisher exact test, as appropriate. Missing data were determined to be missing at random and were addressed through multiple imputations by chained equations employing a random forest algorithm, generating 10 imputed data sets.

Given the observational study design and numerous confounders across the 3 THV groups, inverse probability of treatment weighting (IPTW)^14^ was applied to address potential bias. Propensity scores estimating each patient's likelihood of undergoing TAVR with either the Evolut, Acurate, or Portico/Navitor valves were calculated using covariate balancing propensity score weighting^15^ across all imputed data sets, targeting the average treatment effect. Covariates were selected based on clinical relevance and observed differences across the 3 valve groups and included: baseline characteristics (year of TAVR, age at TAVR, sex, body mass index), comorbidities (hypertension, diabetes mellitus, chronic kidney disease [estimated glomerular filtration rate ≤60 mL/min/1.73 m^2^], atrial fibrillation, chronic pulmonary disease, peripheral vascular disease), medical history (previous myocardial infarction, previous percutaneous coronary intervention, previous cerebrovascular incident, prior pacemaker implantation), echocardiographic features (aortic valve area, mean aortic valve gradient, maximum aortic valve gradient, aortic annulus diameter, systolic pulmonary artery pressure, bicuspid/tricuspid aortic valve, ejection fraction, moderate/severe mitral insufficiency, moderate/severe aortic insufficiency), laboratory values, and clinical features (N-terminal prohormone of brain natriuretic peptide, New York Heart Association functional class III or IV) as well as procedural characteristics (access site, urgency of TAVR procedure).

The generated weights were Winsorized at the 97th percentile to mitigate the influence of extreme weights. The standardized mean difference (SMD) was used to evaluate the covariate balance between valve groups, with an SMD of less than 10% indicating an acceptable balance.

The impact of THV type on all-cause mortality was assessed using weighted Cox regression models. Unadjusted and IPTW-adjusted Kaplan-Meier survival curves were generated by weighting the survival function.

Marginal effects for each outcome were estimated using a doubly robust approach combining the weights from IPTW and G-computation, with the Evolut valves as the reference group.^16^ Post-TAVR mean aortic valve gradients were treated as a continuous variable and estimated using a weighted linear regression model with the generated weights. Subsequently, the Games-Howell test was applied to adjust for multiple comparisons. Confidence intervals for all outcomes were calculated using robust standard errors with the sandwich estimator.

All *p* values were two-sided, with values below 0.05 considered statistically significant. Analyses were conducted in R (R Foundation for Statistical Computing, version 4.3.1, Vienna, Austria).

## Results

### Baseline Characteristics

The study cohort consisted of 1068 patients, with the most frequently implanted valve type being the Evolut (n ​= ​488), followed by the Acurate (n ​= ​478) and the Portico/Navitor valves (n ​= ​102) (Table 1). The mean ± SD European system for cardiac operative risk evaluation (EUROSCORE) II-predicted mortality varied significantly between the valve types, being the highest in Portico/Navito (5.3% ​± ​4.5%) compared to Evolut (4.5% ​± ​4.4%) and Acurate valves (4.1% ​± ​3.7%) (*p* ​< 0.001). Patients receiving Portico/Navitor valves were also older (83.0 ​± ​6.0 years) than those receiving Acurate (81.4 ​± ​6.5) or Evolut (80.5 ​± ​7.3) valves (*p* ​< ​0.001). Most patients were female (88%), with no significant difference in sex distribution among valve groups (*p* ​= ​0.10).

**Table 1:** Baseline characteristics of the TAVR population with small aortic annulus

Characteristic	Evolut N ​= ​488∗	Acurate N ​= ​478∗	Portico/Navitor N ​= ​102∗	*p* value†	Missing
Age (y)	80.5 ± 7.3	81.4 ± 6.5	83.0 ± 6.0	**<0.001**	0
Sex				0.10	0
Male	53 (11%)	66 (14%)	7 (6.9%)		
Female	435 (89%)	412 (86%)	95 (93%)		
EUROSCORE II predicted mortality (%)	4.5 (4.4)	4.1 (3.7)	5.3 (4.5)	**0.02**	0
BMI (kg/m^2^)	26.6 ± 5.7	26.1 ± 5.2	25.0 ± 4.7	**0.02**	9
NYHA functional class 3 or 4	384 (79%)	300 (63%)	85 (83%)	**<0.001**	0
NTproBNP (ng/dL)	3129.4 ± 5294.4	2606.1 ± 4902.7	3693.6 ± 5868.1	0.2	152
Concomitant diseases					
Hypertension	387 (79%)	353 (74%)	74 (73%)	0.09	0
Diabetes mellitus	105 (22%)	108 (23%)	23 (23%)	>0.9	0
CKD (eGFR <60 mL/min/1.73 m^2^)	274 (56%)	246 (52%)	68 (67%)	**0.02**	5
Atrial fibrillation	138 (28%)	154 (32%)	37 (36%)	0.2	0
Chronic pulmonary disease	92 (19%)	82 (17%)	16 (16%)	0.7	0
Peripheral vascular disease	83 (17%)	53 (11%)	11 (11%)	**0.02**	0
Previous history					
Previous myocardial infarction	21 (4.3%)	7 (1.5%)	1 (1.0%)	**0.02**	0
Previous PCI	125 (26%)	78 (16%)	19 (19%)	**0.002**	0
Previous cerebrovascular incident	59 (12%)	38 (7.9%)	7 (6.9%)	0.06	0
Previous pacemaker	53 (11%)	35 (7.3%)	10 (11%)	0.14	12
Echocardiographic features and valve characteristics					
Mean aortic valve gradient (mmHg)	49.6 ± 14.1	48.6 ± 12.6	53.5 ± 12.8	**0.002**	7
Maximum aortic valve gradient (mmHg)	80.7 ± 21.4	79.4 ± 19.6	86.5 ± 19.1	**0.005**	95
Aortic valve area (cm^2^)	0.7 ± 0.2	0.7 ± 0.1	0.6 ± 0.1	**0.003**	253
Aortic valve annular diameter (mm)	22.0 ± 1.0	22.0 ± 1.0	22.0 ± 0.9	0.7	0
Pulmonary artery systolic pressure (mmHg)	26.6 ± 13.1	27.2 ± 12.6	30.4 ± 13.7	0.07	243
Moderate or severe aortic regurgitation	55 (12%)	40 (8.5%)	8 (8.2%)	0.3	21
Moderate or severe mitral regurgitation	51 (11%)	60 (13%)	15 (15%)	0.4	21
Bicuspid aortic valve	29 (6.4%)	14 (3.0%)	2 (2.2%)	**0.03**	57
Left ventricular ejection fraction				0.8	28
≥50%	392 (82%)	396 (85%)	83 (86%)		
40%-49%	50 (11%)	45 (9.6%)	8 (8.2%)		
<40%	34 (7.1%)	26 (5.6%)	6 (6.2%)		

Abbreviations: BMI, body mass index; CKD, chronic kidney disease; eGFR, estimated fglomerular filtration rate; EUROSCORE, European system for cardiac operative risk evaluation; NTproBNP, N-terminal prohormone of brain natriuretic peptide; NYHA, New York Heart Association; PCI, percutaneous coronary intervention; TAVR, transcatheter aortic valve replacement.

The values in bold represent differences between the groups with *p* value < 0.05.

∗ n (%); mean ± SD.

† Welch’s F test (Welch’s analysis of variance); Pearson's chi-squared test; Fisher exact test.

Among comorbidities, peripheral vascular disease was more common in the Evolut group (17%, *p* ​= ​0.02). Chronic kidney disease (estimated glomerular filtration rate ≤60) was more prevalent in the Portico/Navitor group (67%) than in the Acurate (52%) and Evolut (56%) groups (*p* ​= ​0.02).

The prevalence of prior myocardial infarction within 3 months and previous percutaneous coronary intervention was higher in the Evolut group. No significant differences were found in rates of hypertension, diabetes mellitus, prior stroke, atrial fibrillation, chronic pulmonary disease, or prior pacemaker implantation (Table 1).

### Echocardiographic Features

Patients receiving Portico/Navitor valves had the highest mean aortic valve gradient (53.5 ​± ​12.8 mmHg vs 49.6 ​± ​14.1 in Evolut and 48.6 ​± ​12.6 in Acurate, *p* ​= ​0.002) and the smallest valve area (0.6 ​± ​0.1 cm^2^, *p* ​= ​0.003). Bicuspid valves were more prevalent in the Evolut group (6.4%) compared to Acurate (3.0%) and Portico/Navitor (2.2%) (*p* ​= ​0.03). Left ventricular ejection fraction distribution was similar across groups (*p* ​= ​0.8), with 82% of patients having left ventricular ejection fraction ≥50%.

### Procedural Characteristics

There were no significant differences in procedural urgency, with 87% of patients undergoing elective TAVR. Transfemoral access was used most frequently across all groups (91% Evolut, 99% Acurate, 99% Portico/Navitor; *p* ​< ​0.001). Predilatation was nearly universal (92%), while postdilatation varied significantly among groups (*p* ​< ​0.001), with the lowest rates being in Evolut valves (23%). 46% of all the valves were oversized by ​≥ ​15%, and this was more prominent in the Evolut group (78%) (Table 2).

**Table 2:** Procedural characteristics according to TAVR valve

Characteristic	Overall N ​= ​1,068∗	Evolut N ​= ​488∗	Acurate N ​= ​478∗	Portico/Navitor N ​= ​102∗	*p* value†	Missing
TAVR urgency					0.14	0
Elective	930 (87%)	432 (89%)	415 (87%)	83 (81%)		
Urgent	138 (13%)	56 (11%)	63 (13%)	19 (19%)		
Access site					<0.001	0
Transfemoral	1019 (95%)	444 (91%)	474 (99%)	101 (99%)		
Via subclavian artery	44 (4.1%)	42 (8.6%)	1 (0.2%)	1 (1.0%)		
Transapical	3 (0.3%)	0 (0%)	3 (0.6%)	0 (0%)		
Direct aortic access	2 (0.2%)	2 (0.4%)	0 (0%)	0 (0%)		
Prosthesis size (mm)					<0.001	0
23	293 (27%)	23 (4.7%)	258 (54%)	12 (12%)		
25	268 (25%)	0 (0%)	193 (40%)	75 (74%)		
26	390 (37%)	390 (80%)	0 (0%)	0 (0%)		
27	41 (3.8%)	0 (0%)	27 (5.6%)	15 (14%)		
29	76 (7.1%)	75 (15%)	0 (0%)	0 (0%)		
Oversizing by ​≥ ​15%‡	491 (46%)	382 (78%)	72 (15%)	37 (36%)	<0.001	0
Predilatation	978 (92%)	402 (82%)	475 (99%)	101 (99%)	<0.001	0
Postdilatation	381 (36%)	110 (23%)	233 (49%)	38 (37%)	<0.001	0

Abbreviation: TAVR, transcatheter aortic valve replacement.

∗ n (%).

† Pearson's chi-squared test; Fisher exact test.

‡ Theoretical device oversizing was defined using the following calculation: ([valve diameter−annulus diameter] × 100)/annulus diameter.

### Inverse Probability of Treatment Weighting

**Figure 2:**
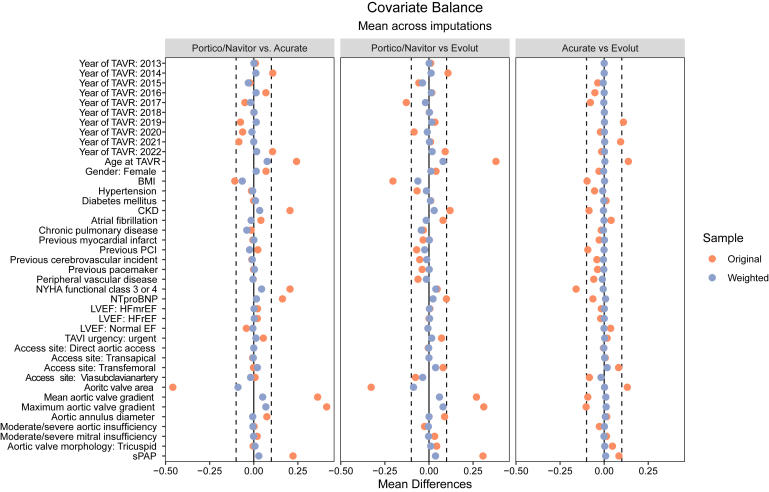
Standardized mean differences across covariates before and after inverse probability of treatment weighting. The dotted lines represent standardized mean differences of ±0.10. Abbreviations: BMI, body mass index; CKD, chronic kidney disease; EF, ejection fraction; HFrEF, heart failure with reduced ejection fraction; HFmrEF, heat failure with mildly reduced ejection fraction; LVEF, left ventricular ejection fraction; NTproBNP, N-terminal prohorme of brain natriuretic peptide; NYHA, New York Heart Association; PCI,percutaneous coronary intervention; sPAP, systolic pulmonary artery pressure; TAVI, transcathter aortic valve implantation; TAVR, transcatheter aortic valve replacement.

Figure 2 and Supplementary Table 2 display the SMDs in covariates before and after applying IPTW. The largest residual difference was observed for the aortic valve area, with an absolute SMD of 0.09 for both Acurate valves vs Portico/Navitor and Evolut vs Portico/Navitor. The second largest difference was the age at TAVR, with an absolute SMD of 0.08 in both Acurate valves vs Portico/Navitor and 0.08 for Evolut vs Acurate valves comparisons. Overall, IPTW achieved an effective balance, with all covariates showing SMDs of or below 0.10.

### Outcomes

Outcomes are presented in Table 3. Periprocedural mortality was observed in 1.2% of Evolut patients (n = 6), 0.2% in the Acurate group (n ​= ​1), and 1.0% in the Portico/Navitor group (n ​= 1). Compared to Evolut, the adjusted odds ratio (aOR) of periprocedural mortality was similar for Acurate (aOR: 0.99; 95% CI: 0.97-1.01; *p* ​= ​0.3) and marginally lower for Portico/Navitor (aOR: 0.99; 95% CI: 0.98-1.00; *p* ​= ​0.05).

**Table 3:** Unadjusted and IPTW-adjusted risk of outcomes

Outcome∗	n/N	Event rate (%) (95% CI)	Unadjusted OR/HR† (95% CI)	*p* value	IPTW-adjusted OR/HR‡ (95% CI)	*p* value
30-d all-cause mortality						
Evolut	6/488	1.2 (0.6%-2.7%)	-	-	-	-
Acurate	1/478	0.2 (0.0%-1.2%)	0.13 (0.02-0.73)	**0.02**	0.99 (0.97-1.01)	0.3
Portico/Navitor	1/102	1.0 (0.2%-5.3%)	0.09 (0.009-0.82)	**0.03**	0.99 (0.98-1.00)	**0.05**
All-cause mortality at median follow-up of 969 d						
Evolut	128/488	26.2 (22.5%-30.3%)	-	-	-	-
Acurate	90/478	18.8% (15.6%-22.6%)	0.84 (0.63-1.09)	0.2	0.86 (0.58-1.28)	0.5
Portico/Navitor	34/102	33.3% (24.9%-42.9%)	1.52 (1.04-2.20)	**0.03**	1.26 (0.70-2.26)	0.4
Technical success						
Evolut	443/488	90.8 (87.9%-93.0%)	-	-	-	-
Acurate	444/478	92.9 (90.2%-94.9%)	1.32 (0.89-1.96)	0.2	1.32 (0.71-2.46)	0.4
Portico/Navitor	90/102	88.2 (80.6%-93.1%)	0.54 (0.29-1.01)	**0.05**	0.54 (0.21-1.41)	0.2
Device success						
Evolut	382/488	78.3 (74.4%-81.7%)	-	-	-	-
Acurate	392/478	82.0 (78.3%-85.2%)	1.38 (1.00-1.93)	**0.05**	1.39 (0.94-2.04)	0.1
Portico/Navitor	75/102	73.5 (64.2%-81.1%)	0.74 (0.45-1.21)	0.2	0.74 (0.40-1.36)	0.3
Mean gradient >20 mmHg or peak >40 mmHg post-TAVR						
Evolut	13/488	2.7% (1.6%-4.5%)	-	-	-	-
Acurate	8/478	1.7% (0.9%-3.3%)	0.69 (0.33-1.45)	0.3	0.71 (0.24-2.06)	0.5
Portico/Navitor	0/102	0.0% (0.0%-3.6%)	-	-	-	-
More than mild PVL at discharge						
Evolut	18/488	3.7% (2.3%-5.8%)	-	-	-	-
Acurate	12/478	2.5% (1.4%-4.3%)	0.63 (0.24-1.69)	0.3	0.63 (0.18-2.20)	0.5
Portico/Navitor	4/102	3.9% (1.5%-9.7%)	1.24 (0.61-2.51)	0.5	1.28 (0.28-5.70)	0.7
Pacemaker following TAVR						
Evolut	40/488	8.2% (6.1%-11.0%)	-	-	-	-
Acurate	33/478	6.9% (5.0%-9.5%)	0.85 (0.45-1.61)	0.6	0.85 (0.39-1.84)	0.7
Portico/Navitor	10/102	9.8% (5.4%-17.1%)	1.58 (0.94-2.67)	0.08	1.58 (0.61-4.12)	0.3

*Notes.* Results reported as number of events (n), event rate %, 95% CI, HR, OR (95% CI), and *p* values. Evolut serves as the reference group.

The values in bold represent differences between groups with *p* < 0.05.

Abbreviations: HR, hazards ratio; IPTW, inverse probability of treatment weighting; OR, odds ratio; PVL, paravalvular leakage; TAVR, transcatheter aortic valve replacement.

∗ Supplementary Table 1 for outcome definitions.

† Generated with univariable logistic analysis/Cox regression analysis. HR was analyzed via Cox regression analysis for the outcome all-cause mortality (at a median follow-up of 969 d). All other outcomes had OR assessed via logistic regression analysis.

‡ Generated with IPTW-adjusted logistic modeling/Cox regression analysis.

Significant differences in all-cause mortality for the median follow-up time of 996 days (IQR: 478-1452 days) were initially observed among the unadjusted patient groups, with those receiving Portico/Navitor valves showing higher mortality rates compared to patients receiving Evolut valves (hazard ratio [HR]: 1.52; 95% CI: 1.04-2.20; *p* ​= ​0.03). However, these differences were not statistically significant after IPTW adjustment (adjusted HR Acurate 0.86; 95% CI: 0.58-1.28; *p* ​= ​0.5, adjusted HR Portico/Navitor 1.26; 95% CI: 0.70-2.26; *p* ​= ​0.4).

**Figure 3:**
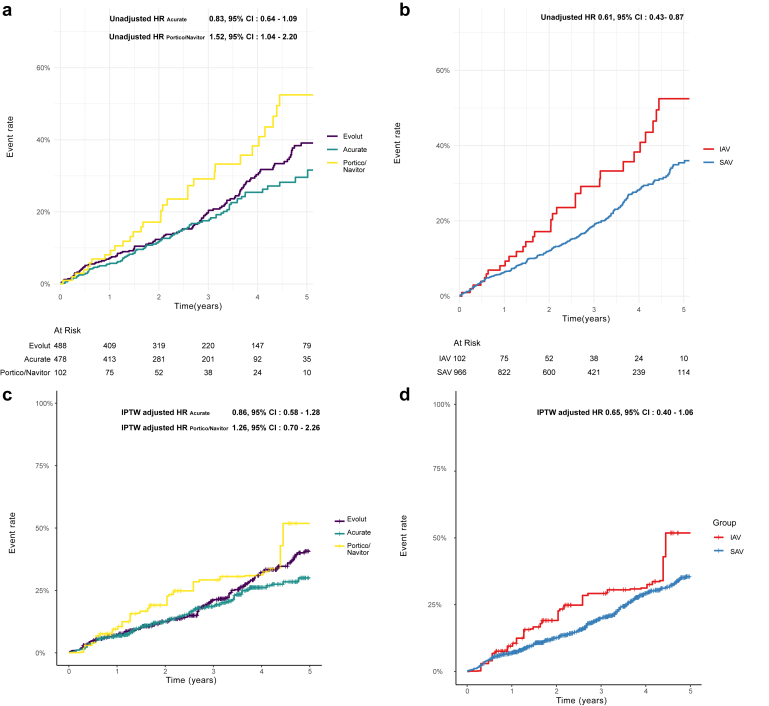
Nonadjusted (Panel a and b) and IPTW-adjusted Kaplan-Meier curves (Panel c and d) of all-cause mortality in patients with small aortic annulus. At a median follow-up of 969 days (IQR: 478-1452 days), Abbott/Portico valves were associated with higher unadjusted mortality compared to Evolut valves (Panel a); however, this difference was not statistically significant after IPTW adjustment (Panel c). No significant differences in mortality were observed between Acurate and Evolut valves in either the unadjusted or IPTW-adjusted analyses. SAVs demonstrated lower unadjusted mortality compared to IAVs (Panel b), but this difference was not significant after IPTW adjustment (Panel d). Note: Number at risk is not applicable for IPTW-adjusted analyses. Abbreviations: HR, hazard ratio; IAV, intra-annular valve; IPTW, inverse probability of treatment weighting; IQR, interquartile range; SAV, supra-annular valve.

Subsequent Kaplan-Meier time-to-event analysis indicated comparable outcomes for all-cause mortality across the different valve manufacturers (Figure 3).

Technical success was high across all valve types: Acurate (93%), Evolut (91%), and Portico/Navitor (88%). After adjustment, no statistically significant differences were observed (Acurate aOR: 1.32; 95% CI: 0.71-2.46; *p* ​= ​0.4; Portico/Navitor aOR: 0.54; 95% CI: 0.21-1.41; *p* ​= ​0.2) (Table 3, Supplementary Table 3). Device success was also comparable between groups, with an aOR for patients with Acurate valves of 1.39 (95% CI: 0.94-2.04; *p* ​= ​0.1) and an aOR for Portico/Navitor of 0.74 (95% CI: 0.40-1.36; *p* ​= ​0.3) (Table 3 and Supplementary Table 4).

The incidence of patients with a mean post-TAVR gradient over 20 mmHg or a peak gradient over 40 mmHg was rare and comparable (Acurate 1.7%, Evolut 2.7%, Portico/Navitor 0%) with no significant differences. Similarly, the incidence of more than mild PVL at discharge was 2.5% in Acurate valves (n ​= ​12) compared to Evolut valves (n ​= 18, 3.7%), with no statistically significant difference noted (aOR: 0.63; 95% CI: 0.18-2.20; *p* ​= ​0.5). Portico/Navitor valves (n ​= 4, 3.9%) showed an equal likelihood of PVL relative to Evolut ones (aOR: 1.28; 95% CI: 0.28-5.70; *p* ​= ​0.7).

There were no statistically significant differences in the outcome of pacemaker need following TAVR among the 3 groups (aOR Acurate 0.85; 95% CI: 0.39-1.84; *p* ​= ​0.7, and aOR Portico/Navitor 1.58; 95% CI: 0.61-4.12; *p* ​= ​0.3).

**Figure 4:**
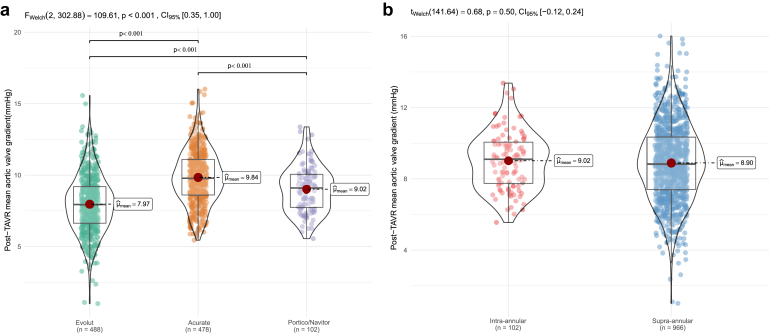
Mean post-TAVR aortic valve gradients across different manufacturers (a) and according to THV valve position (b). Abbreviations: TAVR, transcatheter aortic valve replacement; THV, transcatheter heart valve.

Post-TAVR mean aortic valve gradients differed significantly across THV types after IPTW adjustment (Figure 4). Evolut valves showed the lowest mean gradient (7.97 mmHg), followed by Portico/Navitor (9.02 mmHg) and Acurate (9.84 mmHg). Overall differences were significant (Welch’s F-test; *p* ​< ​0.001; 95% CI: 0.35-1.00), with post hoc comparisons confirming significant differences between all valve pairs (*p* ​< ​0.001). When stratified by valve position, no significant difference was observed between SA (8.90 mmHg) and IA valves (9.02 mmHg; *p* ​= ​0.5; 95% CI: −0.12 to 0.24).

## Discussion

This study aimed to compare clinical outcomes and hemodynamic performance among different SEVs in patients with small aortic annulus (SAA), utilizing registry data from 1068 patients. No statistically significant differences were observed in all-cause mortality (median follow-up: 996 days, IQR: 478-1452), technical success, or device success. The only observed difference was a marginally lower rate of periprocedural mortality with the IA SEV Portico/Navitor compared to the SA SEVs Evolut. However, this finding is likely due to chance or residual confounding rather than a true clinical difference.

In addition, no significant differences were observed in the rates of more than mild PVL at discharge or the requirement for a pacemaker among the 3 valve types. Regarding hemodynamic outcomes, the Evolut valves produced the lowest postprocedural mean aortic valve gradients, followed by the Portico/Navitor valves, while the Acurate valves were associated with the highest postprocedural mean gradients. Whether these differences reflect inherent valve design characteristics remains uncertain. It is worthwhile to note that Evolut and Portico/Navitor valves were more frequently oversized compared to Acurate, resulting in the use of larger THV sizes, which may partly explain the lower gradients observed in these groups.

Patients with SAA represent up to one-third of those with aortic stenosis, with a notable predominance of women.^3^^,^^17^^,^^18^ This subgroup is at increased risk for compromised valve hemodynamics, including elevated post-TAVR mean aortic gradients, prosthesis-patient mismatch (PPM), reduced exercise capacity, and diminished prosthesis durability.^17^^,^^19^, ^20^, ^21^ As such, there is a growing interest in studies comparing the different THVs in these patients, their clinical outcomes, and their hemodynamic performance.

Most previous studies have concentrated on comparing SEVs to BEVs in patients with SAA. Results from 3 independent registries—BERN TAVI,^22^ OPERA TAVI,^23^ and transcatheter aortic valve implantation for small aortic annuli (TAVI SMALL 2)^24^—demonstrate that, in SAA patients, SEV implantation is associated with a more favorable hemodynamic profile than BEV implantation. The SMART^10^ trial remains the only randomized study comparing a SEV (Evolut) and a BEV (SAPIEN) in patients with severe aortic stenosis and SAA. At 12 months, the SEV patients demonstrated noninferior clinical outcomes compared to the BEV patients, with similar rates of mortality, disabling stroke, and rehospitalization for heart failure. However, Evolut valves outperformed SAPIEN valves on multiple hemodynamic measures, with a larger effective orifice area and a significantly lower rate of severe PPM. It also exhibited fewer cases of bioprosthetic valve dysfunction at 1 ​year, which may translate into greater durability.

The reasons behind the superior hemodynamic performance of SEVs remain unclear; some attribute it primarily to the SA design, while others suggest it may be due to the continuous expansion properties of the nitinol frame in these valves. Consequently, similar hemodynamic performance might be anticipated from the IA SEV Portico/Navitor, comparable to that of its SA SEV counterparts.

Studies comparing SA SEVs and IA SEVs in this specific population remain limited. To our knowledge, only one prior study has examined this, using propensity score matching in a cohort of 86 matched pairs.^25^ This study reported no significant differences in clinical outcomes, including periprocedural, early, and late mortality, device success, technical success, clinical efficacy, 2-year survival, or 2-year stroke incidence. Furthermore, hemodynamic performance outcomes—such as 30-day and 1-year residual mean gradients and rates of PPM—were similar between SA and IA SEVs.

Our study findings are largely consistent with those of the previously mentioned study. Therefore, our study does not support the superiority of SA SEVs over IA SEVs in terms of clinical outcomes. However, unlike the prior study, we identified significant differences in postprocedural mean gradients. Portico/Navitor valves, while associated with higher postprocedural gradients than Evolut valves, demonstrated lower gradients than Acurate valves. This finding supports the theory that the self-expanding mechanism, rather than the SA design, contributes to lower postprocedural gradients. Interestingly, while the TAVR SMALL study^17^ reported higher gradients in IA SEVs compared to SA ones, our study did not find such an association. However, this may be influenced by the smaller patient population in the Portico/Navitor group, which could limit the ability to detect differences.

The hemodynamic performance of the valve following aortic valve replacement is critical for ensuring both short- and long-term outcomes.^26^^,^^27^ In this real-world data set, all 3 examined THV types demonstrated excellent hemodynamic profiles. Although differences were noted in postprocedural mean aortic valve gradients, these variations are minor, and their clinical significance remains uncertain. Other factors, such as coronary access and the possibility of a second TAVR procedure if needed, may be of more importance when selecting a THV device for an individual patient. A randomized study comparing short- and long-term outcomes across different SEVs is, therefore, highly warranted.

## Limitations

Despite rigorous statistical methods such as IPTW and multivariable regression being employed to account for baseline differences between groups, these methods adjust only for observed covariates. Therefore, residual confounding due to unmeasured or unknown factors may still influence the results. Although to a lesser extent, missing data were present in several variables. While multiple imputation using chained equations was employed to handle these missing values, the impact that the true, unobserved values may have had on the assessment of procedural, baseline characteristics, and IPTW remains unknown. Additionally, we lacked information on the medications used by each patient, and as a result, we were unable to adjust for these in our analysis.

## Data Availability Statement

The data sets utilized in this article are subject to Swedish privacy and confidentiality laws, restricting public access.

## Ethics Statement

This study was conducted in accordance with the principles of the Declaration of Helsinki, and it was approved by the Swedish Ethical Review Authority.

## Funding

A. Louca has received funds through the 10.13039/501100005754Sahlgrenska University Hospital funds (SU-984277) and the Gothenburg Society of Medicine (1000894 and 1021054).

## Disclosure Statement

A. Louca reports financial support was provided by Sahlgrenska University Hospital and Göteborg Medical Society. The other authors had no conflicts to declare.
