# Supplementary material for: Self-Expandable Transcatheter Aortic Valves in Patients With Small Aortic Annulus: The SWEDEHEART Registry
Source: Struct Heart. 2025 Jun 18;9(11):100680. doi: 10.1016/j.shj.2025.100680 (PMC12766495; doi:10.1016/j.shj.2025.100680)
Supplement: Supplementary Table 1 [file mmc1.docx]

**Supplementary Table 1. Outcome definitions**

| **Technical success**  **(at exit from procedure room)** | - Freedom fr**om mortality** - Successful access, delivery of the device, and retrieval of the delivery system - Correct positioning of a single prosthetic heart valve into the proper anatomical location - Freedom from surgery or intervention related to the device or to a major vascular or access-related, or cardiac structural complication |
| --- | --- |
| **Device success**  **(during index hospitalisation)** | - Technical success - Freedom fr**om mortality** - Freedom from surgery or intervention related to the device or to a major vascular or access-related or cardiac structural complication - Intended performance of the valve (mean gradient <20 mmHg, peak velocity <3 m/s, Doppler velocity index ≥0.25, and less than moderate aortic regurgitation) |
| Reproduced by the Valve Academic Research Consortium 3: Updated Endpoint Definitions for Aortic Valve Clinical Research | |
