# Supplementary material for: Self-Expandable Transcatheter Aortic Valves in Patients With Small Aortic Annulus: The SWEDEHEART Registry
Source: Struct Heart. 2025 Jun 18;9(11):100680. doi: 10.1016/j.shj.2025.100680 (PMC12766495; doi:10.1016/j.shj.2025.100680)
Supplement: Supplementary Table 2 [file mmc2.docx]

**Table 3. Covariate balance after IPTW**

|  | Boston vs Abbott | | Medtronic vs Abbott | | Medtronic vs Boston | |
| --- | --- | --- | --- | --- | --- | --- |
| **Variable** | **Unadjusted SMD** | **IPTW-adjusted SMD** | **Unadjusted SMD** | **IPTW-adjusted SMD** | **Unadjusted SMD** | **IPTW-adjusted SMD** |
| Year of TAVI: 2013 | 0.01 | 0.00 | 0.00 | 0.00 | 0.01 | 0.00 |
| Year of TAVI: 2014 | 0.11 | 0.01 | 0.00 | 0.00 | 0.11 | 0.01 |
| Year of TAVI: 2015 | -0.06 | -0.04 | -0.04 | -0.01 | -0.02 | -0.03 |
| Year of TAVI: 2016 | 0.02 | 0.01 | -0.05 | 0.00 | 0.07 | 0.01 |
| Year of TAVI: 2017 | -0.13 | -0.02 | -0.08 | 0.00 | -0.05 | -0.02 |
| Year of TAVI: 2018 | 0.00 | 0.00 | 0.00 | 0.00 | 0.00 | 0.00 |
| Year of TAVI: 2019 | 0.03 | 0.02 | 0.11 | 0.00 | -0.08 | 0.01 |
| Year of TAVI: 2020 | -0.08 | -0.01 | -0.02 | 0.00 | -0.06 | -0.01 |
| Year of TAVI: 2021 | 0.01 | 0.00 | 0.09 | 0.00 | -0.08 | 0.00 |
| Year of TAVI: 2022 | 0.09 | 0.02 | -0.01 | 0.00 | 0.11 | 0.02 |
| Age at TAVI | 0.38 | 0.08 | 0.14 | 0.00 | 0.24 | 0.08 |
| Sex: Female | 0.04 | 0.01 | -0.03 | 0.00 | 0.07 | 0.01 |
| BMI | -0.20 | -0.06 | -0.10 | 0.00 | -0.11 | -0.07 |
| Hypertension | -0.07 | -0.01 | -0.05 | -0.01 | -0.01 | -0.01 |
| Diabetes mellitus | 0.01 | 0.01 | 0.01 | 0.00 | 0.00 | 0.01 |
| CKD | 0.12 | 0.03 | -0.09 | 0.00 | 0.21 | 0.03 |
| Atrial fibrillation | 0.08 | -0.02 | 0.04 | 0.00 | 0.04 | -0.01 |
| Chronic pulmonary disease | -0.03 | -0.04 | -0.02 | 0.00 | -0.01 | -0.04 |
| History of myocardial infarction | -0.03 | 0.00 | -0.03 | 0.00 | 0.00 | 0.00 |
| Previous PCI | -0.07 | -0.02 | -0.09 | 0.00 | 0.02 | -0.02 |
| History of cerebrovascular incident | -0.05 | -0.01 | -0.04 | 0.00 | -0.01 | -0.01 |
| Pacemaker | -0.04 | 0.00 | -0.04 | 0.00 | 0.00 | 0.00 |
| Peripheral vascular disease | -0.06 | -0.01 | -0.06 | -0.01 | 0.00 | 0.00 |
| NYHA functional class III or IV | 0.05 | 0.04 | -0.16 | -0.01 | 0.21 | 0.04 |
| NTproBNP | 0.10 | 0.02 | -0.06 | 0.01 | 0.16 | 0.02 |
| LVEF: HFmrEF | 0.01 | 0.00 | -0.02 | 0.00 | 0.02 | 0.00 |
| LVEF: HFrEF | 0.00 | 0.00 | -0.02 | 0.00 | 0.02 | 0.00 |
| LVEF: Normal EF | -0.01 | -0.01 | 0.04 | 0.00 | -0.04 | 0.00 |
| Aortic valve area | -0.33 | -0.09 | 0.13 | 0.00 | -0.46 | -0.09 |
| Mean aortic valve gradient | 0.27 | 0.06 | -0.09 | 0.01 | 0.36 | 0.05 |
| Maximum aortic valve gradient | 0.31 | 0.08 | -0.10 | 0.01 | 0.41 | 0.07 |
| Aortic annulus diameter | 0.09 | 0.00 | 0.01 | 0.01 | 0.07 | -0.01 |
| sPAP | 0.31 | 0.04 | 0.08 | 0.01 | 0.22 | 0.03 |
| Moderate or severe aortic valve insufficiency | -0.03 | 0.00 | -0.03 | 0.00 | 0.00 | 0.00 |
| Moderate or severe mitral valve insufficiency | 0.03 | 0.00 | 0.01 | 0.00 | 0.02 | 0.00 |
| Aortic valve morphology: Tricuspid | 0.04 | 0.01 | 0.05 | 0.00 | 0.00 | 0.01 |
| TAVI urgency: Urgent | 0.07 | 0.02 | 0.02 | 0.00 | 0.05 | 0.01 |
| Access site: Transfemoral | 0.08 | 0.04 | 0.08 | 0.02 | 0.00 | 0.02 |
| Access site: Transapical | 0.00 | 0.00 | 0.00 | 0.00 | 0.00 | 0.00 |
| Access site: Via subclavian artery | -0.08 | -0.04 | -0.08 | -0.02 | 0.01 | -0.02 |
