# Supplementary material for: Self-Expandable Transcatheter Aortic Valves in Patients With Small Aortic Annulus: The SWEDEHEART Registry
Source: Struct Heart. 2025 Jun 18;9(11):100680. doi: 10.1016/j.shj.2025.100680 (PMC12766495; doi:10.1016/j.shj.2025.100680)
Supplement: Supplementary Table 3 [file mmc3.docx]

**Supplementary Table 3:** Comparison of Selected Outcomes for Technical Success Failure Between Different valve manufacturers

| **Characteristic** | **Medtronic**  N = 488*^1^* | **Boston**  N = 478*^1^* | **Abbott**  N = 102*^1^* |
| --- | --- | --- | --- |
| Deceased in procedure room | 0 (0%) | 0 (0%) | 0 (0%) |
| Procedure failure  (Operator assessment) | 14 (3%) | 7 (1%) | 3 (3%) |
| Cardiac tamponade | 2 (0.4%) | 2 (0.4%) | 1 (1.0%) |
| Major bleeding | 16 (3.3%) | 16 (3.3%) | 7 (6.9%) |
| Vascular complication |  |  |  |
| Aorta | 0 (0%) | 0 (0%) | 1 (1.0%) |
| Access site | 10 (2.0%) | 16 (3.3%) | 3 (2.9%) |
| Annulus rupture | 0 (0%) | 0 (0%) | 0 (0%) |
| Other | 1 (0.2%) | 0 (0%) | 0 (0%) |
| Conversion to heart operation | 0 (0%) | 0 (0%) | 0 (0%) |
| Need for vascular intervention | 5 (1.0%) | 7 (1.5%) | 1 (1.0%) |
| Coronary occlusion | 1 (0.2%) | 0 (0%) | 0 (0%) |
| Additional valve needed | 7 (1.4%) | 2 (0.4%) | 5 (4.9%) |
| Valve embolization |  |  |  |
| To aorta | 6 (1.2%) | 0 (0%) | 1 (1.0%) |
| To left ventricle | 0 (0%) | 0 (0%) | 1 (1.0%) |
| *^1^*n (%) | | | |
