# Supplementary material for: Self-Expandable Transcatheter Aortic Valves in Patients With Small Aortic Annulus: The SWEDEHEART Registry
Source: Struct Heart. 2025 Jun 18;9(11):100680. doi: 10.1016/j.shj.2025.100680 (PMC12766495; doi:10.1016/j.shj.2025.100680)
Supplement: Supplementary Table 4 [file mmc4.docx]

Supplementary Table 4. Comparison of Selected Outcomes for Device Failure during Index Hospitalization between Different Valve Manufacturers

| **Outcome** | **Medtronic**  N = 488*^1^* | **Boston**  N = 478*^1^* | **Abbott**  N = 102*^1^* |
| --- | --- | --- | --- |
| Technical failure | 45 (9.2%) | 34 (7.1%) | 12 (12%) |
| Death during index hospitalization | 0 (0%) | 0 (0%) | 0 (0%) |
| Cardiac tamponade | 5 (1.0%) | 3 (0.6%) | 0 (0%) |
| Major bleeding | 16 (3.3%) | 16 (3.3%) | 7 (6.9%) |
| Vascular complication | 8 (1.6%) | 13 (2.7%) | 4 (3.9%) |
| More-than-mild PVL | 18  (3.7%) | 12  (2.5%) | 4  (3.9%) |
| Prosthesis-patient mismatch | 13  (2.7%) | 8  (1.7%) | 0  (0%) |
| Myocardial infarction | 1 (0.2%) | 1 (0.2%) | 0 (0%) |
| *^1^*n (%) | | | |
